# Prediction Model for Mechanical Properties of Lightweight Aggregate Concrete Using Artificial Neural Network

**DOI:** 10.3390/ma12172678

**Published:** 2019-08-22

**Authors:** Jin Young Yoon, Hyunjun Kim, Young-Joo Lee, Sung-Han Sim

**Affiliations:** School of Urban and Environmental Engineering, Ulsan National Institute of Science and Technology, Ulsan 44919, Korea

**Keywords:** artificial neural network, compressive strength, elastic modulus, lightweight aggregate concrete, prediction model

## Abstract

The mechanical properties of lightweight aggregate concrete (LWAC) depend on the mixing ratio of its binders, normal weight aggregate (NWA), and lightweight aggregate (LWA). To characterize the relation between various concrete components and the mechanical characteristics of LWAC, extensive studies have been conducted, proposing empirical equations using regression models based on their experimental results. However, these results obtained from laboratory experiments do not provide consistent prediction accuracy due to the complicated relation between materials and mix proportions, and a general prediction model is needed, considering several mix proportions and concrete constituents. This study adopts the artificial neural network (ANN) for modeling the complex and nonlinear relation between constituents and the resulting compressive strength and elastic modulus of LWAC. To construct a database for the ANN model, a vast amount of detailed and extensive data was collected from the literature including various mix proportions, material properties, and mechanical characteristics of concrete. The optimal ANN architecture is determined to enhance prediction accuracy in terms of the numbers of hidden layers and neurons. Using this database and the optimal ANN model, the performance of the ANN-based prediction model is evaluated in terms of the compressive strength and elastic modulus of LWAC. Furthermore, these prediction accuracies are compared to the results of previous ANN-based analyses, as well as those obtained from the commonly used linear and nonlinear regression models.

## 1. Introduction

Lightweight aggregate concrete (LWAC) represents a type of concrete which has a low unit weight compared to that of normal weight aggregate concrete (NWAC). Because of the low mass density, LWAC has the advantages associated with reduced self-weight of structures and has been applied in long-span bridges and high-rise buildings [1]. Furthermore, structural LWAC, with a strength that is similar to NWAC, enables the reduction of construction cost as it requires less reinforcement, smaller supporting deck members, beams, and piers, and less earthquake damage. Another important economic benefit of LWAC is the transportation cost savings achieved by improving the lifting efficiency in the construction field and lowering shipping cost, compared to conventional NWAC products. The unique advantages of LWAC allow a wide variety of real world applications that are continuously increasing [2].

LWAC consists of the same concrete components as conventional NWAC with a partial or complete replacement of normal weight aggregate (NWA) with lightweight aggregate (LWA). Commercially available artificial LWAs are generally produced by sintering pyroplastic materials such as slate, shale, clay, and by-products of coal combustion materials at 1200–1300 °C [3]. These LWAs have an inherently great porosity, resulting in low density, low strength, and deformable particles [4,5]. Following ASTM C 330 standard tests [6], these LWAs conform to the specifications of a particle density not exceeding 2000 kg/m^3^, or a dry loose bulk density less than 1120 kg/m^3^, 880 kg/m^3^, and 1040 kg/m^3^ for fine, coarse, and combined LWA, respectively. LWAC incorporating these LWAs generally has a density lower than 1920 kg/m^3^ [7]. A lower density of LWAC can be achieved by using a larger amount of porous LWA, resulting in poor mechanical performance. Because the failure of LWAC is influenced by the friable and deformable LWAs, most LWAC has a lower compressive strength and elastic modulus than NWAC.

Extensive efforts have been made to characterize the relation between LWAC constituents and the mechanical properties of compressive strength and elastic modulus. A previous study showed that the compressive strength and elastic modulus of LWAC were inversely proportional to the volume fraction of LWA [8]. The compressive strength of LWAC also depends on not only the content of LWAs, but also their type [9,10]. Hence, these experimental studies indicated that the properties and amount of LWAs influenced the mechanical behavior of LWAC. Furthermore, the mix proportions of LWAC are also essential parameters influencing the performance of LWAC, such as water-to-cement ratio (w/c) and mass of aggregate, water, and binders including cement, fly ash, and silica fume [11]. Considering these factors, some guidelines were provided for estimating the mechanical properties of LWAC, which were not consistently attainable due to the complexities associated with LWA properties and mix proportions [7,12]. Therefore, the reliable prediction model for mechanical characteristics of LWAC is required to extend its use in the construction with ensuring its performance.

To consider the highly complicated relationship between concrete constituents and properties of cement-based construction materials, researchers have employed artificial neural networks (ANN). An ANN is a numerical model using highly interconnected processing of simple computing elements, referred to as neurons, imitating the structure and functions of biological neural systems of the human brain [13]. In the field of construction materials, ANN methods were applied for modeling concrete properties, including mechanical characteristics, fluidity, and durability using concrete components-related information as input parameters [14,15,16]. Ni and Wang [14] reported that the single-layer ANN model showed a good prediction accuracy for estimating the 28-day compressive strength of NWAC. Note that the ANN models of 11-7-1 from Ni and Wang [14] and 7-5-3-2 from Oztas et al. [16] in Table 1 indicate 11 inputs, seven neurons in one hidden layer, and one output and seven inputs, five and three neurons in each hidden layer, and two outputs, respectively. Douma et al. [15] likewise used a single hidden layer ANN model for the prediction of fluidity and mechanical properties of self-compacting concrete, where results were well-matched to the target value. In the case of LWAC, Alshihri et al. [17] and Tavakkol et al. [18] investigated the ANN-based estimation of compressive strength of LWAC fabricated in the laboratory environment. In addition, the ANN model having a single hidden layer structure was used for predicting the Poisson ratio [19] and compressive strength based on measured ultrasonic pulse velocity [20]. These experimental results provided the feasibility of the ANN to model the nonlinear relation between various parameters and concrete properties. However, as prediction modeling for LWAC has not been fully studied, further research is still desired, to evaluate the optimal ANN architecture and consider extensive data about mix proportions, binders, and aggregates.

This study presents an ANN-based prediction model for mechanical characteristics of LWAC, which enable us to produce high-quality LWAC that satisfies the target performance. First, detailed and extensive data on the mix proportions and the mechanical behavior of LWAC are collected from the literature. The vast amount of data allows to enhance the reliability and accuracy of the prediction model. Input parameters for the ANN model are selected for better modeling of the LWAC, including water-to-binder ratio, amount of binders, density of concrete, and volume fraction of aggregates. Subsequently, the appropriate number of hidden layers and neurons are determined for developing the optimal ANN architectures to obtain high prediction accuracy. Finally, the performance of the ANN-based prediction model is evaluated and compared to the results obtained from the commonly used statistical models.

## 2. Research Background

### 2.1. Artificial Neural Network

**Figure 1:**
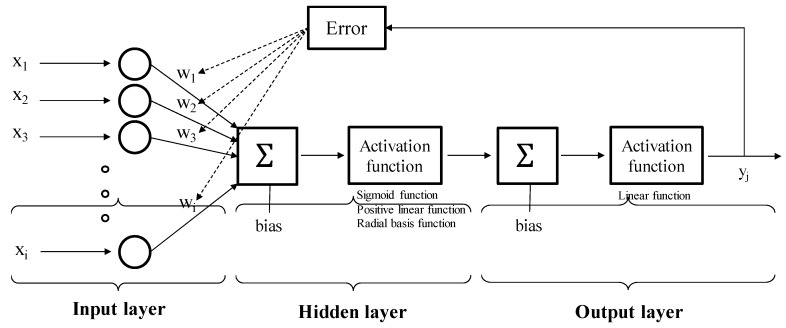
Schematic diagram for artificial neural network [21].

An ANN is a network based on a collection of connected neurons used to model the complex relationship between inputs and outputs. As shown in Figure 1, the basic structure of an ANN consists of three types of layers: Input, hidden, and output layers. Depending on the number of hidden layers, ANN is classified into single- or multi-layer perceptron, which has multi-connected neurons. When neurons in the input layer receive an input, the weighted sums of the inputs are transferred to interconnected neurons in the next layer and evaluated using activation functions [13]. The weight and bias values can be determined as the solutions of the optimization problem to minimize the prediction error using the back-propagation algorithm [15].

To optimize the weight and bias, the back-propagation learning is repeated from the output layer back to the input layer in each running step, until there is no further decrease in the mean square error (*MSE*) of the prediction. After the training is completed with updated weight and bias, new inputs from the test dataset are used in the network to produce the corresponding outputs. The prediction accuracy can be evaluated by the mean absolute percentage error (*MAE*) and correlation coefficient (*R*). *MSE*, *MAE,* and *R* are defined as:

(1) $$MSE=\frac{1}{N}\sum {\left(y_{i}-y_{di}\right)}^{2}$$

(2) $$MAE=\frac{1}{N}\sum \frac{\left|y_{i}-y_{di}\right|}{\left|y_{i}\right|}$$

(3) $$R=\frac{\sum \left(y_{i}-{\bar{y}}_{i}\right)\left(y_{di}-{\bar{y}}_{di}\right)}{\sqrt{\sum {\left(y_{i}-{\bar{y}}_{i}\right)}^{2}\sum {\left(y_{di}-{\bar{y}}_{di}\right)}^{2}}}$$

where *N* is the number of training data, $y_{i}$ is the target value, $y_{di}$ is the predicted result, ${\overset{¯}{y}}_{i}$ is the mean prediction value, and ${\overset{¯}{y}}_{di}$ is the mean target value. The correlation coefficient is introduced to evaluate the linear dependence between the predicted and target values. Its value ranges from −1 to 1, where 1 indicates the perfect positive correlation and 0 means no linear relation among variables.

### 2.2. ANN-Based Prediction of Concrete Properties

Previous ANN-based prediction models demonstrated the feasibility for predicting physical and mechanical properties of concrete, as provided in Table 1. The most conventionally used ANN-based prediction mainly focused on the characterization of properties of NWAC, such as the compressive strength, elastic modulus, drying shrinkage, and fluidity [14,22,23,24,25]. As these ANN models exhibited acceptable prediction accuracy, their applications were extended to high strength concrete, self-consolidating concrete, recycled aggregate concrete, and LWAC. However, further research on these types of concrete is still needed to enhance the reliability and the prediction accuracy of ANN-based analysis considering various mix proportions and concrete materials. Furthermore, determining optimal numbers of hidden layers and neurons for the ANN model is necessary to model a complex relationship between the inputs and targets. Considering these aspects, this study mainly focuses on the prediction of mechanical properties of LWAC using the ANN model.

In the application of the ANN model, several issues have been identified with regard to the database, input variables, and ANN architecture. First, many studies used the data obtained in the laboratory environment, resulting in an acceptable prediction error [14,17,18]. However, the applications of these ANN models are limited to the concrete using specific types of materials. To make a more general ANN-based prediction model, this study collects various data of LWAC mixtures with different mix proportions and concrete materials provided in Section 3. The selection of appropriate input parameters is another important factor to consider for obtaining high prediction accuracy. Considering the input variables used in previous studies and the unique material properties of LWAC, the inputs are determined as shown in Section 4.1. Lastly, the investigation for designing optimal ANN architecture for predicting mechanical properties of LWAC is necessary in terms of the numbers of hidden layers and neurons. Previous studies, listed in Table 1, used various ANN models with one- and two-hidden layers with 3–29 hidden neurons. As an example, 11-7-1 in Ni and Wang [14] depicts 11 inputs, seven neurons in one hidden layer, and one output ANN architecture. Because these experimental studies attained a good prediction accuracy, this study conducts the ANN analysis using the ANN model with the range of these hidden layers and neurons, as provided in Section 4.2. Finally, vast data, suitable input variables, and optimal ANN architecture are used for the ANN-based prediction model for LWAC.

## 3. Establishment of A Database

Accurate and reliable prediction using the ANN model generally depends on the amount and quality of available data. This study conducted a literature review to collect extensive and detailed information on mix proportions, physical properties of composites, and mechanical characteristics of concrete. A total of 149 concrete mixtures (20 NWAC and 129 LWAC) produced with various mix proportion and aggregates were prepared for ANN-based compressive strength analysis [2,4,8,10,27,28,29,30,31,32,33,34,35,36,37]. For the prediction of the static elastic modulus, data from 133 concrete mixtures (20 NWAC and 113 LWAC) were collected [2,4,8,10,27,28,32,33,34,35,36,37]. The obtained data are summarized in Table 2 listing concrete density (kg/m^3^), water-to-binder ratio (w/b), content of water (kg/m^3^), cement (kg/m^3^), fly ash (kg/m^3^), silica fume (kg/m^3^), coarse NWA (CNWA, kg/m^3^), fine NWA (FNWA, kg/m^3^), coarse LWA (CLWA, kg/m^3^), fine LWA (FLWA, kg/m^3^), volume fraction of aggregate, and the specific gravity of used materials. Detailed information on concrete mixtures is provided in the Appendix A. Note that NWAC is included in the database as control concrete samples without CLWA and FLWA. Compared to the mix proportion of LWAC, the selected data of NWAC have a similar w/b ratio, cement, fly ash, and silica fume content. The variables of mix proportion and volume fraction provided in Table 2 are generally considered as factors influencing the mechanical behavior of concrete [17].

Because several studies used different sizes of specimens for measuring the compressive strength and elastic modulus, the size effect needs to be considered to assure data consistency. The size effect generally presents a reduction of compressive strength and the elastic modulus of concrete with an increasing the height-to-width ratio caused by failure of the heterogeneous concrete composite [38]. Therefore, the correction factors are used to convert the data of compressive strength and elastic modulus to equivalent values for the widely used 100 mm by 200 mm cylindrical specimen, referring to the empirical equations from previous studies [38,39,40,41]. Here, the correction factors for 28-day compressive strength are 1.051 for *f_c,__Ø100__×200_*/*f_c,150__×150×150_*, 0.874 for *f_c,__Ø100__×200_*/*f_c,100__×100×100_*, and 1.230 for *f_c,__Ø100__×200_*/*f_c,__Ø150__×300_* obtained from w/c = 0.49 high performance lightweight foamed concrete [42]. The correction equation for the static elastic modulus is *E_c,Ø__100__×200_* = (*E_c,__Ø150__×300_* − 1.9)/0.9 obtained from normal strength NWAC [41]. Because the investigations of size and shape effect for LWAC were rare, the empirical equations from foamed concrete and normal strength NWAC are used considering the similar failure mechanism induced by the concrete heterogeneity and internal defects of voids. Using these correction factors for the compressive strength and elastic modulus, the database used for the ANN model is updated.

## 4. Prediction Model for Compressive Strength and Elastic Modulus Using ANN

Because the ANN model has been used for modeling highly nonlinear and complex interactions between input and output variables, the selection of appropriate input parameters and the ANN architecture is critical for reliable and accurate prediction. This section mainly illustrates the selection of input factors and the determination of the optimal ANN architecture for the prediction of mechanical characteristics of LWAC.

### 4.1. Input Parameters

The selection of appropriate input variables is an important task for enhancing the prediction accuracy of the ANN model, considering the relation between input and target values. Because the strong correlation between mechanical properties of concrete and the amount of constituent materials is generally known, these parameters were adopted as the input for predicting compressive strength and the elastic modulus using ANN models, as summarized in Table 3 [15,16,17,26]. Commonly used inputs are w/b ratio and mass of water, binders, aggregates, and chemical admixture, which are likewise adopted in this study. However, other parameters such as the sand-to-aggregate ratio, aggregate-to-binder ratio, replacement ratio of binders and aggregates, grade of cement, maximum size of aggregates, and the fine modulus of sand are not included despite their possible relation with mechanical properties, because of the difficulties in data collection, as in [14,43]. Even though previous studies listed in Table 3 provide useful information about the selection of appropriate inputs, NWAC was of primary interest in the prediction. Therefore, additional variables tailored to LWAC are needed to improve the prediction performance.

Accordingly, this study adopts the density of LWAC and volume fraction of aggregates as the inputs due to the inverse proportional relation to the mechanical performance of LWAC [44]. Because the linear and nonlinear empirical relation between the density of LWAC and compressive strength or elastic modulus has been reported, the use of this value as an input parameter is expected to enhance the prediction accuracy [45,46]. Furthermore, the volume fractions of aggregates are introduced as an input parameter regarding the amount of LWA in the mixture, replacing the mass. The volume fraction that reflects the actual volume of added LWA can be viewed as another indicator of how much LWA is contained in the mixture in addition to the mass. Thus, the volume fractions are included as the input variables for NWA for comparison.

In summary, the selected input variables used for the ANN model are w/b ratio, concrete density, mass of water, cement, fly ash, silica fume, and volume fraction of CNWA, FNWA, CLWA, and FLWA as provided in Table 3. In the analysis of compressive strength and elastic modulus, these inputs are adopted for the ANN-based prediction. 

### 4.2. Determination of the Optimal ANN Architecture

Determination of the optimal ANN architecture in terms of the number of hidden layers and neurons is the most fundamental part of ANN-based analysis. An excessive number of hidden neurons can cause an over-fit problem due to overestimation in the complex relationship between inputs and targets. An insufficient number of hidden neurons can cause an underfitting problem due to the lack of neurons covering various inputs. Therefore, this study determines the optimal ANN architecture with the minimum *MSE* value, conducting the tests with consideration to various numbers of hidden layers and neurons. The testing ranges of the numbers of hidden layers and neurons are selected by referring the proposed optimization methodologies and previously applied ANN models. First, many optimized methodologies have been proposed to find appropriate numbers of hidden neurons in the higher-order ANN. Among these methods, three empirical equations are selected to estimate the number of neurons in hidden layers, considering the numbers of inputs and outputs in the ANN models [47,48,49]. The equations are provided as

(4) $$N_{h}=\frac{\sqrt{1+8N_{i}}-1}{2}$$

(5) $$N_{h}=N_{i}-1$$

(6) $$N_{h}=\frac{4N_{i}^{2}+3}{N_{i}^{2}-8}$$

where *N_h_* and *N_i_* are the number of hidden neurons and inputs, respectively. Because a total of 10 input parameters are selected in the previous section, the corresponding numbers of calculated hidden neurons are 4, 9, and 4.4 from Equations (4)–(6), respectively. Furthermore, the architectures of previously applied ANN models (see Table 1) are considered to select the appropriate testing ranges. The applied ANN architectures had a higher number of neurons ranging from 3–29 with one or two hidden layers. Thus, to determine the optimal ANN architecture, this study considers the number of neurons from one to 30, which includes the recommended values from Equations (4)–(6) as well as the used ones in Table 1. In the case of hidden layers in the ANN model, previous studies adopted one- or two- hidden layer ANN models, which showed acceptable prediction accuracy. To evaluate the performance of multi-hidden layer ANN models, this study selects a wide range of hidden layers from 1 to 10.

To determine the optimal ANN architecture with the lowest prediction *MSE* for testing data, detailed information of the ANN model is provided regarding the backpropagation algorithm, activation function, five-fold cross validation, and used computing resources. This study employs the scaled conjugate gradient backpropagation as a network training scheme that updates weight and bias values according to the scaled conjugate gradient method provided in the MATLAB Deep Learning Toolbox [50]. A nonlinear hyperbolic tangent sigmoid function is employed as an activation function in the hidden layer, and a linear transfer function is used in the output layer. To enhance the reliability of the ANN model, five-fold cross validation is used [51,52]. Once five sets of approximately equal-sized samples are prepared through the random partition process, a subset of each sample is used to fit the ANN model, and the remaining samples evaluate the prediction accuracy of the model. This is repeated five times. The network error is the average error obtained from the five-fold cross validation method providing greater confidence in the error evaluation. The evaluation of prediction accuracy is conducted using the following computing resources: Intel(R) Core i5-6600 ~3.3 GHz (4 CPUs), NVIDIA GeForce GTX 1080, and 8 GB RAM. 

**Figure 2:**
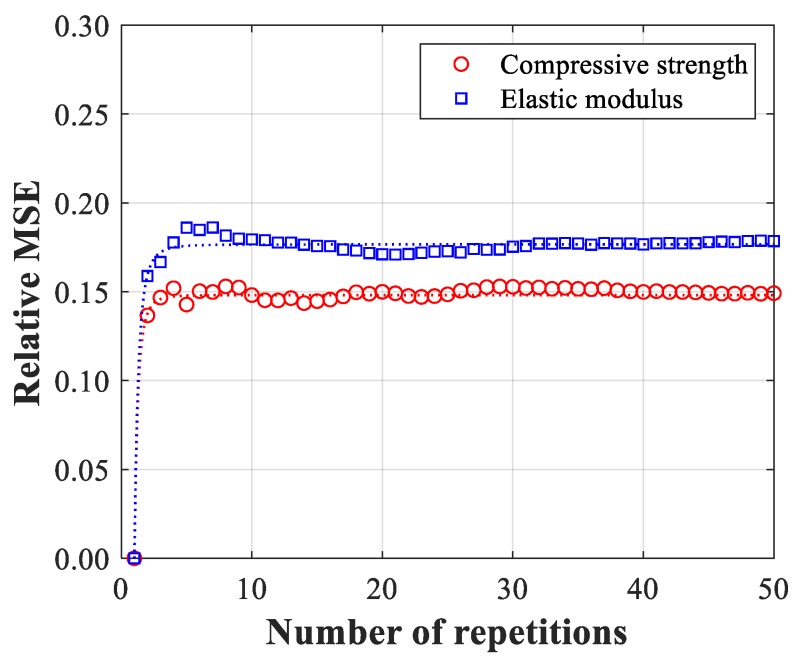
Relative *MSE* for compressive strength and elastic modulus.

In the determination of the optimal ANN architecture, the averaged *MSE* obtained is used for reliable ANN-based prediction. To show the convergence of averaged *MSE* values, the relative *MSE* defined as (*MSE*(1) − *MSE*(*i*))/*MSE*(1) is calculated for 50 repetitions, as shown in Figure 2. The results of the relative *MSE* show a convergence of averaged *MSE* for multiple repetitions, compared to the result of *MSE*(1). Due to the high computational cost during the test, the appropriate number of repetitions needs to be selected based on the regression curve from the relative *MSE* of compressive strength and elastic modulus, marked as “Comp” and “Els” in Figure 2, respectively. The regression curve is −0.148*x*^−3.812^ + 0.148 for compressive strength and −0.176*x*^−3.391^ + 0.176 for the elastic modulus. When the slope of 10^−4^ is selected as a threshold of convergence, the minimum numbers of the required repetitions are 7 and 8 for the compressive strength and elastic modulus, respectively, which yield the corresponding MSE values. 

**Figure 3:**
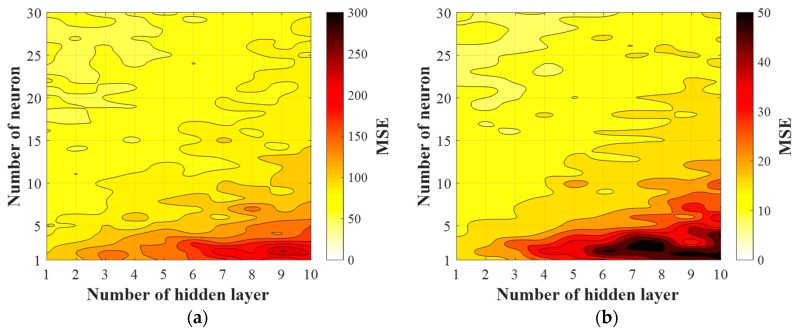

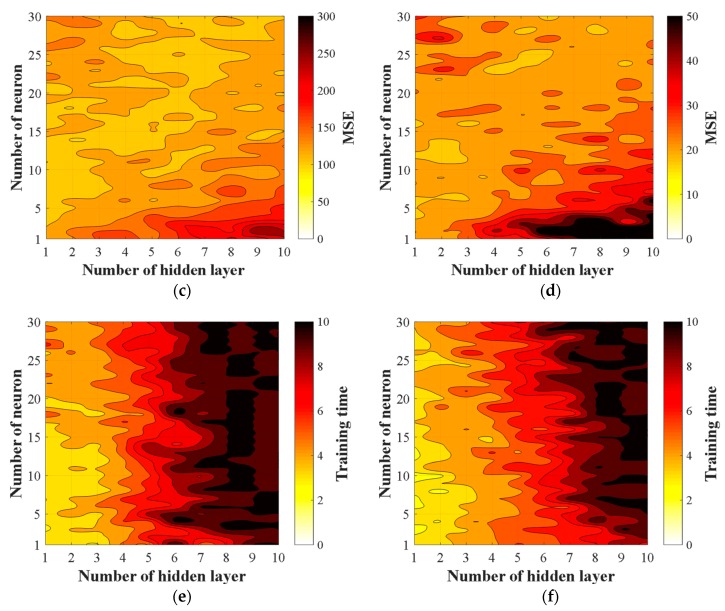
ANN performance evaluation: (**a**) training, (**c**) test, and (**e**) training time for compressive strength and (**b**) training, (**d**) test, and (**f**) training time for elastic modulus analyses.

In the prediction of the compressive strength and elastic modulus, the optimal ANN architecture is determined considering *MSE* values for training and testing and computation time. The *MSE* results for both training and testing are provided in Figure 3 obtained by the ANN models with the 1–30 neurons and 1–10 hidden layers. As expected, the prediction results for both the compressive strength and elastic modulus show much lower *MSE* values in training than in testing. When the optimal architecture of the ANN model is determined at the minimum test *MSE*, as provided in Table 4, two hidden layers with 14 neurons in each hidden layer for compressive strength analysis show the best performance with the training and a test *MSE* of 48.7 and 98.2, respectively. For the elastic modulus analysis, the minimum training and test *MSE* values are 7.8 and 16.9, respectively, corresponding to four hidden layers and 23 neurons. The training times depicted in Figure 3e,f show that a greater number of hidden layers for given number of neurons increases the computational cost significantly due to more numerical calculation. An overfitting problem at small numbers of hidden neurons and many hidden layers is related to the vanishing gradient problem due to slower optimization in earlier layers than in later layers [53].

## 5. Evaluation of Prediction Accuracy

Using the input variables and optimal ANN architecture determined in the previous section, the performance of the ANN-based prediction model is evaluated with respect to the compressive strength and elastic modulus of LWAC. Furthermore, the prediction results are compared to those obtained from the linear and nonlinear regression models using the same input data.

### 5.1. Prediction Results Using the ANN Model

**Figure 4:**
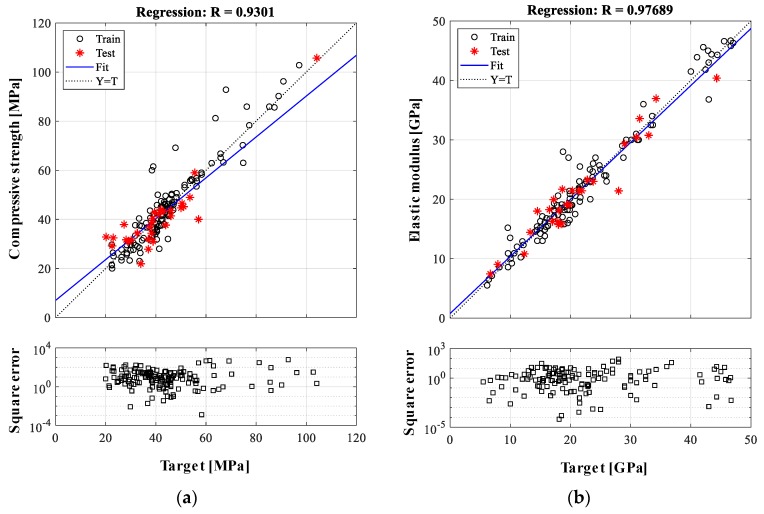
Prediction results from ANN model: (**a**) compressive strength and (**b**) elastic modulus.

Prediction accuracies for compressive strength and elastic modulus of LWAC are evaluated using the selected inputs and ANN architectures with respect to the square error, *MAE*, and correlation coefficient. Figure 4 shows the ANN-based prediction results with the square errors of each input data, when the ANN models have two hidden layers and 14 neurons, and four layers and 23 neurons for estimation of the compressive strength and elastic modulus, respectively. For the compressive strength analysis, the square error ranges from 1.3 × 10^−3^ to 6.2 × 10^2^ and from 3.6 × 10^−2^ to 2.9 × 10^2^ for training and testing, respectively. *MAE* values are 9.6% and 14.5% for training and testing, respectively. Furthermore, 87% of the testing data has *MAE* values of less than 30%, while the correlation coefficient of the predicted and reference data is 0.930. Prediction results for the elastic modulus show better accuracy. The square error ranges from 1.5 × 10^−4^ to 8.5 × 10 for training and from 6.5 × 10^−5^ to 4.4 × 10 for testing. *MAE* values are 6.9% and 8.5% for training and testing, respectively, which depicts results that are much lower than those for the compressive strength. In addition, the square error of the individual testing data does not exceed a *MAE* of 30%, resulting in a high correlation coefficient of 0.977. Note that the large variations of square error for compressive strength are derived from its raw data of compressive strength having larger variation than that of the elastic modulus. These prediction errors obtained from the ANN models are summarized in Table 5. Compared to the prediction accuracy of the ANN model for NWAC, less than 12.4% of *MAE*, reported by previous studies listed in Table 1, the present ANN-based model for LWAC exhibits acceptable performance for predicting the compressive strength and elastic modulus.

### 5.2. Comparative Analysis

In Section 5.1, the prediction performance of the ANN model is validated, and the results are compared to those obtained from the commonly used regression algorithms: The statistical models of linear and nonlinear regression. These regression models have typically been used for modeling the relationship between constituents and concrete properties [18,45,46]. Thus, this study adopts the linear and nonlinear (i.e., second-order polynomial) regression models for the evaluation of prediction performance. Furthermore, their prediction accuracies are compared to those obtained from the ANN models using scale-independent *MAE*.

**Figure 5:**
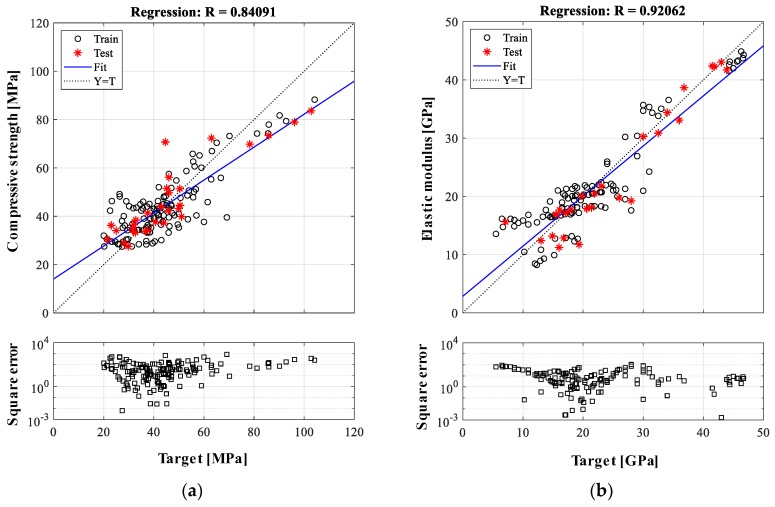
Prediction results from multiple linear regression (MLR)-based analysis: (**a**) compressive strength and (**b**) elastic modulus.

In the linear regression model, multiple linear regression (MLR) is employed to identify the relationship between two or more independent variables and the dependent variable. The equation for the generally used MLR model is provided as

(7) $$y=\alpha_{0}+\alpha_{1}x_{1}+\alpha_{2}x_{2}+\cdots +\alpha_{i}x_{i}$$

where y depicts the properties of LWAC being predicted, $x_{i}$ depicts the independent variables, $\alpha_{0}$ is the *y*-intercept, and $\alpha_{i}$ is the regression coefficient. Using the same input variables and data randomly divided into an 8:2 ratio for training and testing likewise the ANN model, the coefficients are optimized with respect to minimizing the summed square error between predicted and target values. The residuals from MLR model are equally distributed and homoscedastic. Due to the variance of network errors at every analysis, MLR-based predictions are repeated 10 times. The linear regression model with the minimum *MAE* value for test is provided, as shown in Figure 5. The prediction-accuracy indicators for the compressive strength are 87.3, 16.4%, and 0.841 for the *MSE*, *MAE*, and correlation coefficient, respectively. For the elastic modulus, Figure 5b shows the MLR-based prediction as 12.9, 14.4%, and 0.921 for the *MSE*, *MAE*, and correlation coefficient, respectively. The relatively high prediction errors from MLR-based analysis could be related to the assumption that the relationship between the mixture components and concrete properties is linear.

**Figure 6:**
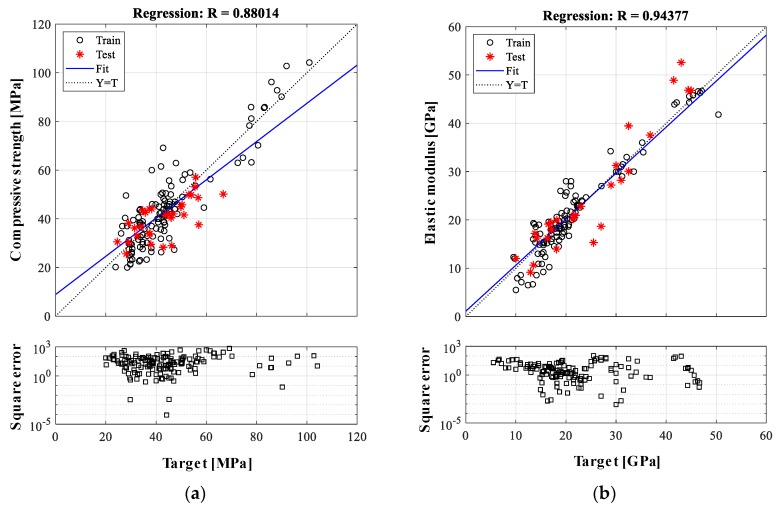
Prediction results from multiple nonlinear regression (MNLR)-based analysis: (**a**) compressive strength and (**b**) elastic modulus.

Considering the complex and nonlinear relationships between the constituents and concrete properties, the nonlinear models employing higher-order polynomials help improve the prediction results. In this study, the multiple nonlinear regression (MNLR) model contains constant, linear, and quadratic terms as follows:

(8) $$y=\alpha_{0}+\alpha_{1}x_{1}+\alpha_{2}x_{2}\cdots +\alpha_{i}x_{i}+\beta_{1}x_{1}^{2}+\beta_{2}x_{2}^{2}+\cdots +\beta_{i}x_{i}^{2}$$

where $\beta_{i}$ is the coefficient for $x_{i}^{2}$. The coefficients of the nonlinear regression model are likewise determined by minimizing the square error between estimated and target values. The same database used in the ANN model is applied for MNLR-based analysis, with an 8:2 ratio for training to testing data quantities. The residuals from MNLR model are also equally distributed and homoscedastic likewise MLR model. The training data optimize the coefficients of the MNLR model, and the prediction performance of this model is evaluated. The prediction results with the minimum prediction error are selected on the basis of the 10 times repetitions, as shown in Figure 6. For the compressive strength prediction, the prediction results are 64.2, 15.0%, and 0.880 for the *MSE*, *MAE*, and correlation coefficient, respectively. Figure 6b shows the results of the elastic modulus with values of 17.6, 13.4%, and 0.944 for the *MSE*, *MAE*, and correlation coefficient. As expected, the quadratic polynomial-based MNLR model demonstrates a better prediction accuracy than the result obtained from the MLR model. Despite the improvement of prediction accuracy using the MNLR model, the ANN-based model shows a better performance.

The prediction accuracies obtained from ANN, MLR, and MNLR models are summarized in Table 6 in terms of the scale-independent *MAE* value. Compared to averaged MLR and MNLR models from 10 times analyses, the ANN-based prediction shows the highest accuracy with *MAE* of 14.5% and 8.5% for the compressive strength and elastic modulus, respectively. High averaged *MAE* values from linear and nonlinear regression models among 10 times repetitions show their wide range variations for predicting mechanical properties of LWAC. These variations in prediction accuracies indicate that used statistical models would not provide the reliable prediction results for LWAC. Thus, the ANN-based prediction model is shown to be suitable for characterizing the complex and nonlinear relationship between the concrete component materials and mechanical characteristics of LWAC.

## 6. Conclusions

This study presents the ANN-based prediction model for the compressive strength and elastic modulus of LWAC. The ANN model considers the complex and nonlinear relation between the mechanical properties and the concrete components of binders, NWAs, and LWAs. The database for the ANN model was constructed by collecting experimental data of various mix proportions, material properties, and mechanical properties of LWAC from the literature. Suitable input parameters and the optimal ANN architecture in terms of the number of hidden layers and neurons were determined to obtain a better prediction accuracy. To enhance the reliability of the ANN model, the five-fold cross validation was applied to all training and validation processes. The scaled conjugate gradient back-propagation algorithm was applied to obtain the optimal weight and bias values in the ANN model during the training stage. Subsequently, the prediction accuracy of the ANN model was evaluated. In comparison to previous studies using the ANN-based prediction model for NWAC, the ANN model used in this study showed acceptable prediction accuracy with respect to the compressive strength and elastic modulus of LWAC. Furthermore, a comparative analysis showed that the highest prediction accuracy is obtained by the ANN model compared to the use of statistical linear and nonlinear regression models.

## Figures and Tables

**Table 1:** Previous studies using artificial neural network (ANN) analysis for predicting mechanical behavior of cement-based material.

Literature	Target	ANN Architecture (Number of Neurons in Input-Hidden-Output Layers)
Ni and Wang (2000) [14]	Compressive strength (65 data)	11-7-1
Oztas et al. (2006) [16]	Compressive strength and fluidity (187 data)	7-5-3-2
Demir (2008) [22]	Elastic modulus (159 data)	1-3-1, 1-5-1, 1-3-3-1
Alshihri et al. (2009) [17]	Compressive strength (108 data)	8-14-4, 8-14-6-4
Atici (2011) [23]	Compressive strength (27 data)	3-5-1, 4-6-1, 4-6-1 3-5-1, 5-6-1, 2-4-1
Bal and Buyle-Bodin (2013) [24]	Drying shrinkage (176 data)	11-8-4-1, 11-8-6-1 11-9-4-1, 11-9-6-1
Khademi et al. (2016) [26]	Compressive strength (257 data)	14-29-1
Douma et al. (2017) [15]	Fluidity (114 data)	6-17-1
Hossain et al. (2018) [25]	Compressive and tensile strength (180 data)	12-8-1, 10-7-1

**Table 2:** Database for ANN analysis.

Mix Proportion and Material Properties		LWAC	NWAC
Concrete density		1170–2280 kg/m^3^	2030–2430 kg/m^3^
w/b		0.23–0.55	0.25–0.45
Mass	Water	150–263 kg/m^3^	158–207 kg/m^3^
	Cement	300–710 kg/m^3^	300–640 kg/m^3^
	Fly ash	0–300 kg/m^3^	0–300 kg/m^3^
	Silica fume	0–71 kg/m^3^	0–96 kg/m^3^
	CNWA	0–810 kg/m^3^	810–1105 kg/m^3^
	FNWA	0–1096 kg/m^3^	288–861 kg/m^3^
	CLWA	0–898 kg/m^3^	0
	FLWA	0–631 kg/m^3^	0
Volume fraction	CNWA	0–0.31	0.30–0.45
	FNWA	0–0.42	0.12–0.34
	CLWA	0–0.52	0
	FLWA	0–0.39	0
Specific gravity	Cement	3100–3160 kg/m^3^	
	Fly ash	2060–2470 kg/m^3^	
	Silica fume	2000–2280 kg/m^3^	
	CNWA	2460–2740 kg/m^3^	
	FNWA	2460–2700 kg/m^3^	
	CLWA	600–2070 kg/m^3^	
	FLWA	1340–1790 kg/m^3^	

**Table 3:** Input variables for the ANN model.

Input Variables	
Oztas et al. [16]	w/b ratio, sand-to-aggregate ratio, replacement ratio of fly ash and silica fume, mass of water, chemical admixture
Alshihri et al. [17]	w/c ratio, curing period, mass of FNWA, CLWA, FLWA, silica fume, chemical admixture
Khademi et al. [26]	w/c ratio, aggregate-to-cement ratio, replacement ratio of recycled aggregate, water-to-total materials ratio, mass of water, cement, CNWA, FNWA, recycled aggregate, chemical admixture
Douma et al. [15]	w/b ratio, replacement ratio of fly ash, content of binders, CNWA, FNWA, chemical admixture
ANN model	w/b ratio, concrete density, mass of water, cement, fly ash, silica fume, volume fraction of CNWA, FNWA, CLWA, FLWA

**Table 4:** Optimal architecture and prediction accuracy of the ANN model.

ANN Model	Prediction for Compressive Strength	Prediction for Elastic Modulus
Number of layers	2	4
Number of neurons	14	23
Training *MSE*	48.7	7.8
Test *MSE*	98.2	16.9

**Table 5:** Prediction accuracy of ANN-based models.

Error Configuration	Compressive Strength		Elastic Modulus	
	Training	Test	Training	Test
Square error	1.3 × 10^−3^–6.2 × 10^2^	3.6 × 10^−2^–2.9 × 10^2^	1.5 × 10^−4^–8.5 × 10	6.5 × 10^−5^–4.4 × 10
*MAE*	9.6%	14.5%	6.9%	8.5%
Correlation coefficient	0.930		0.977	

**Table 6:** Prediction accuracies of ANN, MLR, and MNLR models.

Prediction Accuracy (*MAE*)	Compressive Strength		Elastic Modulus	
	Training	Test	Training	Test
ANN model	9.6%	14.5%	6.9%	8.5%
Linear regression	17.0%	19.7%	19.0%	21.4%
Nonlinear regression	14.3%	19.9%	13.7%	20.1%

## Appendix A

**Table A1:** Data sources.

Literatures	Mix Proportion [kg/m^3^]									Volume Fraction				Density [kg/m^3^]	σ_28_ [MPa]	E_28_ [GPa]
	w/b	W	C	FA	SF	CNWA	CLWA	FNWA	FLWA	CNWA	CLWA	FNWA	FLWA			
Kim et al., 2010. [8]	0.38	175	460	0	0	810	0	861	0	0.30	0	0.34	0	2300	47	37.0
	0.38	175	460	0	0	608	117	861	0	0.22	0.07	0.34	0	2280	44	29.0
	0.38	175	460	0	0	405	234	861	0	0.15	0.15	0.34	0	2220	43	30.0
	0.38	175	460	0	0	203	352	861	0	0.07	0.22	0.34	0	2200	42	33.0
	0.38	175	460	0	0	0	469	861	0	0	0.30	0.34	0	2150	32	30.0
	0.38	175	460	0	0	604	154	861	0	0.22	0.07	0.34	0	2280	44	31.0
	0.38	175	460	0	0	402	308	861	0	0.15	0.15	0.34	0	2150	40	26.0
	0.38	175	460	0	0	201	463	861	0	0.07	0.22	0.34	0	2100	40	24.0
	0.38	175	460	0	0	0	617	861	0	0	0.30	0.34	0	2000	37	25.0
Bogas and Gomes 2013. [30]	0.50	225	450	0	0	0	374	676	0	0	0.35	0.26	0	1763	35	-
	0.50	225	450	0	0	0	303	676	0	0	0.35	0.26	0	1686	27	-
	0.35	158	450	0	0	0	374	847	0	0	0.35	0.32	0	1897	49	-
	0.35	158	450	0	0	0	452	833	0	0	0.35	0.32	0	1942	66	-
	0.35	158	450	0	0	0	247	846	0	0	0.35	0.32	0	1776	31	-
Bogas et al., 2015. [31]	0.55	193	350	0	0	0	382	825	0	0	0.35	0.32	0	1897	38	-
	0.55	193	350	0	0	0	208	825	0	0	0.35	0.32	0	1710	19	-
Nguyen et al., 2014. [4]	0.45	190	426	0	0	0	445	554	0	0	0.45	0.23	0	1440	38	19.3
	0.45	190	426	0	0	0	445	277	213	0	0.45	0.11	0.11	1380	36	18.6
	0.45	190	426	0	0	0	445	0	427	0	0.45	0	0.22	1320	34	17.3
	0.45	190	426	0	0	0	626	554	0	0	0.45	0.23	0	1490	35	19.1
	0.45	190	426	0	0	0	626	277	177	0	0.45	0.11	0.11	1410	33	17.0
	0.45	190	426	0	0	0	626	0	354	0	0.45	0	0.22	1340	31	15.3
	0.45	190	426	0	0	0	558	554	0	0	0.45	0.23	0	1410	31	16.3
	0.45	190	426	0	0	0	558	277	189	0	0.45	0.11	0.11	1290	26	13.6
	0.45	190	426	0	0	0	558	0	379	0	0.45	0	0.23	1170	22	11.1
	0.45	190	426	0	0	0	673	554	0	0	0.45	0.23	0	1520	40	18.2
	0.45	190	426	0	0	0	673	277	189	0	0.45	0.11	0.11	1400	35	15.7
	0.45	190	426	0	0	0	673	0	379	0	0.45	0	0.23	1280	31	13.6
	0.45	190	426	0	0	1105	0	554	0	0.45	0	0.23	0	2030	52	32.7
Choi et al., 2006. [33]	0.38	175	460	0	0	810	0	861	0	0.31	0	0.34	0	2306	49	34.0
	0.38	175	460	0	0	608	117	861	0	0.23	0.07	0.34	0	2221	46	27.0
	0.38	175	460	0	0	405	234	861	0	0.16	0.15	0.34	0	2135	45	24.0
	0.38	175	460	0	0	203	352	861	0	0.08	0.22	0.34	0	2051	46	25.0
	0.38	175	460	0	0	0	469	861	0	0	0.30	0.34	0	1965	34	28.0
	0.38	175	460	0	0	810	0	645	158	0.31	0	0.25	0.10	2248	46	30.0
	0.38	175	460	0	0	810	0	430	316	0.31	0	0.17	0.20	2191	46	33.0
	0.38	175	460	0	0	810	0	215	473	0.31	0	0.08	0.29	2133	53	31.0
	0.38	175	460	0	0	810	0	0	631	0.31	0	0	0.39	2076	59	30.0
Yang and Huang 1998. [34]	0.28	178	626	0	0	0	292	1096	0	0	0.18	0.42	0	2192	41	23.0
	0.28	178	626	0	0	0	299	1096	0	0	0.18	0.42	0	2199	44	23.8
	0.28	178	626	0	0	0	311	1096	0	0	0.18	0.42	0	2211	50	24.7
	0.28	178	626	0	0	0	389	939	0	0	0.24	0.36	0	2132	37	20.6
	0.28	178	626	0	0	0	399	939	0	0	0.24	0.36	0	2142	41	21.5
	0.28	178	626	0	0	0	415	939	0	0	0.24	0.36	0	2158	47	22.6
	0.28	178	626	0	0	0	486	783	0	0	0.30	0.30	0	2073	35	18.2
	0.28	178	626	0	0	0	498	783	0	0	0.30	0.30	0	2085	38	19.0
	0.28	178	626	0	0	0	519	783	0	0	0.30	0.30	0	2106	45	20.3
	0.28	178	626	0	0	0	583	626	0	0	0.36	0.24	0	2013	62	15.8
	0.28	178	626	0	0	0	598	626	0	0	0.36	0.24	0	2028	36	17.2
	0.28	178	626	0	0	0	623	626	0	0	0.36	0.24	0	2053	42	18.7
Kockal and Ozturan 2011. [10]	0.26	158	551	0	55	0	592	636	0	0	0.37	0.24	0	1860	42	19.6
	0.26	157	548	0	55	0	567	633	0	0	0.36	0.24	0	1915	54	26.0
	0.26	157	549	0	55	0	580	634	0	0	0.36	0.24	0	1943	56	25.7
	0.26	158	551	0	55	981	0	636	0	0.36	0	0.24	0	2316	63	36.8
Gesoglu et al., 2007. [36]	0.35	192	550	0	0	0	487	862	0	0	0.27	0.33	0	2101	36	20.0
	0.35	192	547	0	0	0	646	624	0	0	0.36	0.24	0	2015	28	18.0
	0.35	191	547	0	0	0	465	858	0	0	0.27	0.33	0	2070	60	29.0
	0.35	193	550	0	0	0	624	628	0	0	0.36	0.24	0	2000	57	28.0
	0.35	193	550	0	0	0	506	863	0	0	0.28	0.33	0	2122	50	25.0
	0.35	193	550	0	0	0	675	627	0	0	0.38	0.24	0	2056	46	27.0
	0.55	220	399	0	0	0	509	902	0	0	0.29	0.35	0	2032	23	17.0
	0.55	220	401	0	0	0	681	658	0	0	0.38	0.25	0	1960	20	14.0
	0.55	221	403	0	0	0	475	908	0	0	0.28	0.35	0	2010	37	19.0
	0.55	220	399	0	0	0	631	657	0	0	0.37	0.25	0	1907	34	18.0
	0.55	218	397	0	0	0	525	895	0	0	0.29	0.34	0	2038	29	19.0
	0.55	219	399	0	0	0	706	657	0	0	0.39	0.25	0	1981	25	17.0
Chi et al., 2003. [32]	0.28	171	602	0	0	0	297	1096	0	0	0.18	0.42	0	2166	42	22.9
	0.28	171	602	0	0	0	396	939	0	0	0.24	0.36	0	2108	38	21.5
	0.28	171	602	0	0	0	495	783	0	0	0.30	0.30	0	2051	35	20.1
	0.28	171	602	0	0	0	594	626	0	0	0.36	0.24	0	1993	32	18.7
	0.39	202	517	0	0	0	297	1096	0	0	0.18	0.42	0	2112	33	20.3
	0.39	202	517	0	0	0	396	939	0	0	0.24	0.36	0	2054	30	16.5
	0.39	202	517	0	0	0	495	783	0	0	0.30	0.30	0	1997	28	16.5
	0.39	202	517	0	0	0	594	626	0	0	0.36	0.24	0	1939	23	13.8
	0.50	226	453	0	0	0	297	1096	0	0	0.18	0.42	0	2072	30	18.2
	0.50	226	453	0	0	0	396	939	0	0	0.24	0.36	0	2014	26	15.5
	0.50	226	453	0	0	0	495	783	0	0	0.30	0.30	0	1957	23	14.2
	0.50	226	453	0	0	0	594	626	0	0	0.36	0.24	0	1899	21	13.3
	0.28	171	602	0	0	0	304	1096	0	0	0.18	0.42	0	2166	44	22.8
	0.28	171	602	0	0	0	406	939	0	0	0.24	0.36	0	2108	41	21.7
	0.28	171	602	0	0	0	507	783	0	0	0.30	0.30	0	2051	39	18.9
	0.28	171	602	0	0	0	608	626	0	0	0.36	0.24	0	1993	36	18.2
	0.39	202	517	0	0	0	304	1096	0	0	0.18	0.42	0	2112	37	21.4
	0.39	202	517	0	0	0	406	939	0	0	0.24	0.36	0	2054	33	18.2
	0.39	202	517	0	0	0	507	783	0	0	0.30	0.30	0	1997	30	17.4
	0.39	202	517	0	0	0	608	626	0	0	0.36	0.24	0	1939	28	16.1
	0.50	226	453	0	0	0	304	1096	0	0	0.18	0.42	0	2072	27	17.1
	0.50	226	453	0	0	0	406	939	0	0	0.24	0.36	0	2014	26	17.0
	0.50	226	453	0	0	0	507	783	0	0	0.30	0.30	0	1957	25	15.2
	0.50	226	453	0	0	0	608	626	0	0	0.36	0.24	0	1899	22	14.8
	0.28	171	602	0	0	0	317	1096	0	0	0.18	0.42	0	2166	48	23.1
	0.28	171	602	0	0	0	422	939	0	0	0.24	0.36	0	2108	47	21.9
	0.28	171	602	0	0	0	528	783	0	0	0.30	0.30	0	2051	46	20.9
	0.28	171	602	0	0	0	634	626	0	0	0.36	0.24	0	1993	43	19.8
	0.39	202	517	0	0	0	317	1096	0	0	0.18	0.42	0	2112	38	21.9
	0.39	202	517	0	0	0	422	939	0	0	0.24	0.36	0	2054	38	21.4
	0.39	202	517	0	0	0	528	783	0	0	0.30	0.30	0	1997	39	20.6
	0.39	202	517	0	0	0	634	626	0	0	0.36	0.24	0	1939	38	18.0
	0.50	226	453	0	0	0	317	1096	0	0	0.18	0.42	0	2072	31	19.3
	0.50	226	453	0	0	0	422	939	0	0	0.24	0.36	0	2014	30	17.9
	0.50	226	453	0	0	0	528	783	0	0	0.30	0.30	0	1957	28	16.3
	0.50	226	453	0	0	0	634	626	0	0	0.36	0.24	0	1899	30	15.4
Kayali, 2008. [35]	0.27	172	300	300	40	1001	0	288	0	0.37	0	0.12	0	2134	56	32.5
	0.23	150	300	300	40	0	898	0	233	0	0.52	0	0.14	1540	45	16.7
	0.30	193	300	300	40	0	766	0	162	0	0.45	0	0.10	1747	63	23.7
	0.36	207	370	142	57	894	0	626	0	0.33	0	0.24	0	2260	58	32.5
	0.36	207	370	142	57	0	481	0	476	0	0.28	0	0.28	1770	53	19.0
	0.36	207	370	142	57	820	0	626	0	0.33	0	0.24	0	2280	56	31.5
	0.36	207	370	142	57	0	440	0	511	0	0.28	0	0.32	1780	67	25.5
Guneyisi et al., 2012. [29]	0.35	193	550	0	0	0	688	688	0	0	0.36	0.27	0	2124	48	-
	0.35	193	468	83	0	0	677	677	0	0	0.35	0.26	0	2101	45	-
	0.35	193	385	165	0	0	665	665	0	0	0.35	0.26	0	2078	42	-
	0.35	193	523	0	28	0	684	684	0	0	0.36	0.26	0	2117	53	-
	0.35	193	495	0	55	0	680	680	0	0	0.35	0.26	0	2109	54	-
	0.35	193	440	83	28	0	670	670	0	0	0.35	0.26	0	2090	48	-
	0.35	193	413	83	55	0	669	668	0	0	0.35	0.26	0	2085	48	-
	0.35	193	358	165	28	0	661	661	0	0	0.34	0.26	0	2070	43	-
	0.35	193	330	165	55	0	657	657	0	0	0.34	0.25	0	2062	43	-
Rossignolo et al., 2003. [2]	0.34	263	710	0	71	0	447	192	0	0	0.34	0.07	0	1605	54	15.2
	0.37	251	613	0	61	0	494	215	0	0	0.38	0.08	0	1573	50	13.5
	0.41	245	544	0	54	0	533	228	0	0	0.41	0.09	0	1532	46	12.9
	0.45	237	484	0	48	0	560	242	0	0	0.43	0.09	0	1482	43	12.3
	0.49	238	440	0	44	0	585	250.8	0	0	0.45	0.10	0	1460	40	12.0
Aslam et al., 2016. [37]	0.36	173	480	0	0	0	360	890	0	0	0.30	0.33	0	1790	36	7.9
	0.36	173	480	0	0	0	375	890	0	0	0.30	0.33	0	1810	37	9.6
	0.36	173	480	0	0	0	390	890	0	0	0.30	0.33	0	1850	42	10.2
	0.36	173	480	0	0	0	405	890	0	0	0.29	0.33	0	1840	44	11.7
	0.36	173	480	0	0	0	421	890	0	0	0.29	0.33	0	1860	43	13.0
	0.36	173	480	0	0	0	436	890	0	0	0.29	0.33	0	1910	41	15.0
Alengaram et al., 2011. [27]	0.30	179	515	27	54	0	542	436	0	0	0.43	0.16	0	1677	30	7.1
	0.32	187	510	25	50	0	535	430	0	0	0.42	0.16	0	1743	27	6.5
	0.35	201	501	24	48	0	525	422	0	0	0.41	0.16	0	1643	26	5.5
	0.35	189	465	25	50	0	392	784	0	0	0.31	0.29	0	1869	38	10.9
	0.35	205	504	27	54	0	424	637	0	0	0.33	0.24	0	1810	35	10.0
	0.35	216	532	28	56	0	448	560	0	0	0.35	0.21	0	1787	33	8.6
	0.35	229	564	30	60	0	475	475	0	0	0.37	0.18	0	1759	30	7.9
Wee et al., 1996. [28]	0.40	170	425	0	0	1083	0	722	0	0.42	0	0.28	0	2400	63	41.8
	0.40	170	383	0	43	1083	0	722	0	0.42	0	0.28	0	2401	70	43.0
	0.40	170	298	128	0	1083	0	722	0	0.42	0	0.28	0	2401	65	41.5
	0.35	170	437	0	43	1046	0	698	0	0.40	0	0.27	0	2394	86	45.0
	0.35	170	389	0	96	1046	0	698	0	0.40	0	0.27	0	2399	90	44.4
	0.30	165	550	0	0	1045	0	640	0	0.40	0	0.25	0	2400	78	44.3
	0.30	165	495	0	55	1046	0	640	0	0.40	0	0.25	0	2401	86	44.3
	0.30	165	385	165	0	1045	0	640	0	0.40	0	0.25	0	2400	81	43.9
	0.25	160	640	0	0	1043	0	587	0	0.40	0	0.23	0	2430	86	45.6
	0.25	160	608	0	32	1043	0	587	0	0.40	0	0.23	0	2430	96	46.6
	0.25	160	576	0	64	1043	0	587	0	0.40	0	0.23	0	2430	103	46.7
	0.25	160	544	0	96	1043	0	587	0	0.40	0	0.23	0	2430	104	46.3
	0.25	160	448	192	0	1043	0	587	0	0.40	0	0.23	0	2430	93	45.8
